# Successful Colonic Self-Expandable Metal Stent Placement Using a Rotatable Sphincterotome After Conventional Guidewire Insertion Failure: A Case Report

**DOI:** 10.7759/cureus.113185

**Published:** 2026-07-22

**Authors:** Koji Takahashi, Masataka Nakano, Takafumi Sakuma, Hidehiro Kamezaki

**Affiliations:** 1 Gastroenterology, Eastern Chiba Medical Center, Togane, JPN; 2 Pathology, Eastern Chiba Medical Center, Togane, JPN

**Keywords:** colonic stent, colorectal obstruction, guidewire insertion, self-expandable metal stent, sphincterotome

## Abstract

Self-expandable metal stent (SEMS) placement is the standard endoscopic treatment for malignant colorectal obstruction, but its technical success depends on successful guidewire passage, which may fail when a sharply angulated or eccentrically oriented stricture prevents the operator from aligning the catheter tip coaxially with the luminal course. We report a 90-year-old woman with complete transverse colon obstruction caused by an adenocarcinoma near the hepatic flexure, in whom guidewire passage across the tumor was achieved with a rotatable sphincterotome after conventional catheter-based attempts had failed. Despite the use of an angled hydrophilic guidewire, catheter torque, repeated tip re-angulation, and patient repositioning, a standard catheter could not be advanced coaxially into the eccentric residual lumen. A rotatable sphincterotome, whose bending plane can be reoriented circumferentially, allowed the catheter tip to be brought coaxial with the eccentric orifice, so that the guidewire could be advanced across the stricture and a colonic SEMS deployed. The advantage over a conventional catheter or a bare guidewire lay in a steerable, mechanically supported tip that provided directional control aligned with the luminal course. Computed tomography on postprocedure day 1 confirmed resolution of the obstruction, and the patient resumed oral intake the same day. This rotatable sphincterotome, previously applied in biliary and pancreatic procedures, may serve as a rescue tool for technically difficult colonic stenting when the catheter tip cannot be inserted coaxially into the stricture orifice.

## Introduction

Malignant colorectal obstruction is a life-threatening emergency that occurs in approximately 7-29% of patients with colorectal cancer and requires prompt intervention [1,2]. Self-expandable metal stent (SEMS) placement has become the standard endoscopic approach in this setting, serving either as a definitive palliative measure or as a bridge to elective surgery, and thereby reducing the need for emergent colostomy and its associated morbidity [3,4].

Technical success of SEMS placement depends on the ability to advance a guidewire through the obstructing lesion under endoscopic and fluoroscopic guidance. Guidewire passage fails in 10-24% of cases; failure occurs most often when the luminal orifice cannot be adequately visualized en face or when the catheter tip cannot be inserted coaxially with the luminal axis [5-7]. Beyond the risk of technical failure, colonic SEMS placement carries a non-trivial rate of adverse events; the overall complication rate approaches 25%, with reported perforation rates of roughly 9-10%, stent migration of approximately 5-9%, and re-obstruction as a leading cause of late reintervention [4,8,9]. This underscores the importance of first-pass technical success, particularly in high-risk patients who are unsuitable for salvage surgery. Several strategies have been proposed to address difficult guidewire passage, including angled-tip guidewires, patient repositioning, and sphincterotome-assisted guidewire insertion (SAGWI) [10]. The rationale for SAGWI rests on the capacity of the sphincterotome tip to be deflected, allowing the operator to insert the catheter tip coaxially into the orifice of a tortuous or eccentric stricture, an approach not possible with a rigid catheter. Although SAGWI improves technical success in observational studies [10,11], conventional sphincterotomes deflect in only a single plane through blade tension, which limits directional control when the luminal axis of the stricture deviates substantially from the endoscopic line of approach.

A rotatable sphincterotome combining a 360-degree rotatable handle with a dual-action blade mechanism allows the direction of tip deflection to be reoriented circumferentially rather than confined to a single fixed plane. This class of device has been applied in technically demanding biliary and pancreatic procedures [12-14]. Sphincterotome-assisted guidewire insertion is itself an established technique; to our knowledge, however, the use of a rotatable sphincterotome to accomplish colonic SEMS placement has not previously been reported. Here we describe such a case, in which SEMS placement for malignant colorectal obstruction was achieved with a rotatable sphincterotome after conventional catheter-based guidewire insertion had failed, and we discuss the potential role of this device relative to existing rescue techniques.

## Case presentation

A 90-year-old woman with a history of chronic heart failure, paroxysmal atrial fibrillation, and chronic kidney disease was brought to our emergency department 13 days before the endoscopic procedure with sudden-onset dyspnea and generalized weakness. She was admitted to the cardiology ward with acute exacerbation of chronic heart failure and improved gradually with standard medical management. Plain abdominal computed tomography (CT) on admission incidentally revealed a colonic mass suspicious for malignancy; given her advanced age, further workup was initially deferred.

**Figure 1:**
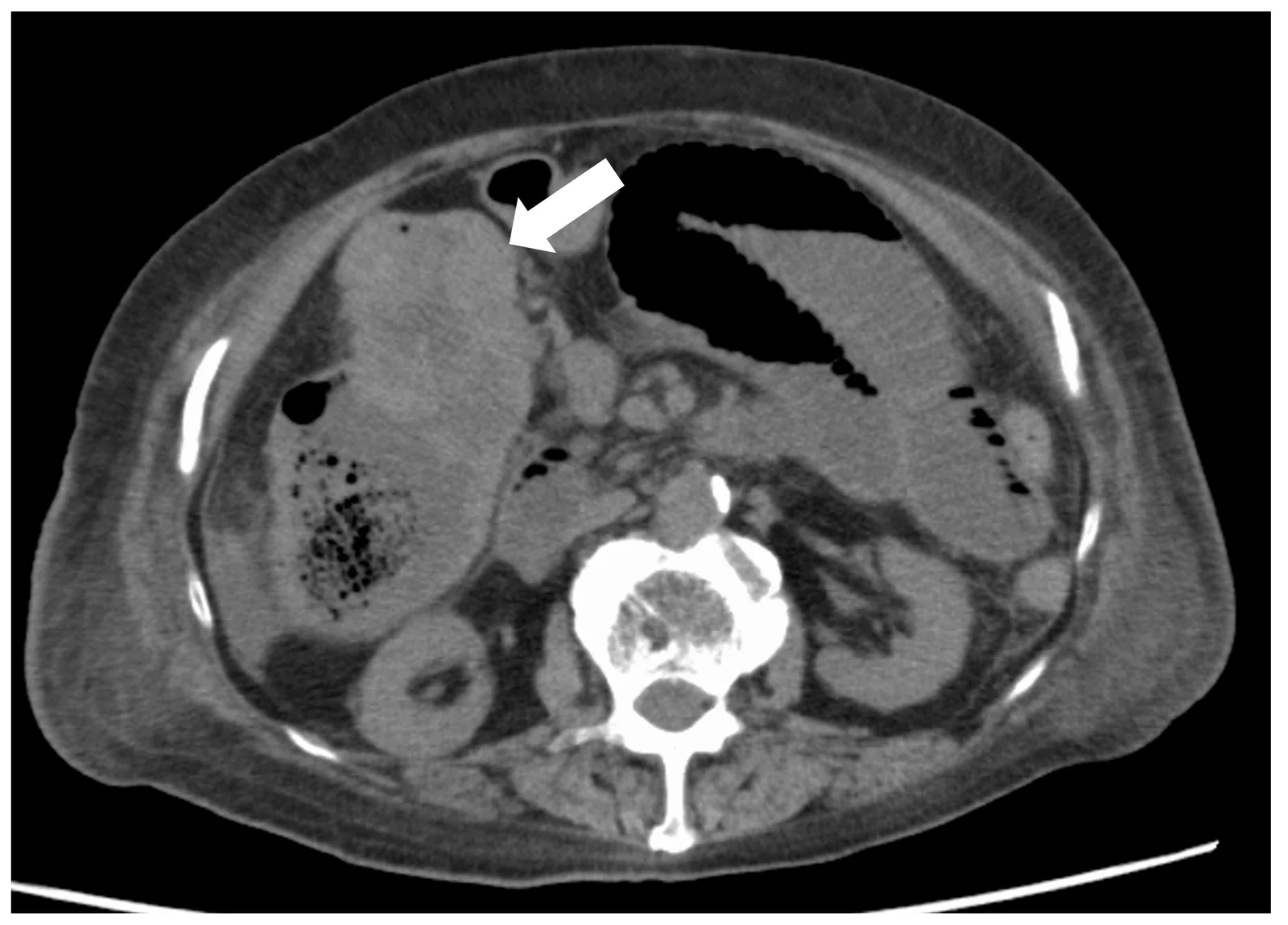
Unenhanced axial computed tomography image of the abdomen obtained on admission. Unenhanced axial computed tomography (CT) image of the abdomen obtained on admission, showing a circumferential mass (arrow) at the hepatic flexure of the transverse colon with marked mural thickening and irregular intraluminal morphology. The lesion caused near-complete large bowel obstruction with pronounced dilatation of the proximal colon. Contrast-enhanced CT was withheld because of underlying chronic kidney disease. No extraluminal gas or free fluid was identified, excluding perforation.

Thirteen days after admission, on the day of endoscopy, she developed persistent poor oral intake and recurrent vomiting, prompting gastroenterology consultation. Repeat CT confirmed a transverse colon tumor near the hepatic flexure causing complete large bowel obstruction with marked proximal colonic distension (Figure 1). Contrast-enhanced CT was withheld because of chronic kidney disease; unenhanced CT showed no obvious distant metastasis. Laboratory investigations revealed marked leukocytosis (white blood cell count 20,590/µL) and elevated C-reactive protein (3.79 mg/dL), consistent with obstructive colitis (Table 1). Given her high surgical risk attributable to cardiac and renal comorbidities, advanced age, and nutritional status, endoscopic SEMS placement was selected as the primary intervention.

**Table 1:** Laboratory findings at the time of endoscopy. ALT: alanine aminotransferase; AST: aspartate aminotransferase; BUN: blood urea nitrogen; CK: creatine kinase; Cl: chloride; CRP: C-reactive protein; K: potassium; LDH: lactate dehydrogenase; MCH: mean corpuscular hemoglobin; MCHC: mean corpuscular hemoglobin concentration; MCV: mean corpuscular volume; Na: sodium; RBC: red blood cell count; WBC: white blood cell count. H: above the upper reference limit; L: below the lower reference limit.

Parameter	Value	Reference range	Unit	Flag
Total protein	6.0	6.6–8.1	g/dL	L
Albumin	2.4	4.1–5.1	g/dL	L
Total bilirubin	0.5	0.4–1.5	mg/dL	
AST	23	13–30	IU/L	
ALT	29	7–23	IU/L	H
LDH	364	125–220	IU/L	H
CK	71	41–153	IU/L	
BUN	21.7	8.0–20.0	mg/dL	H
Creatinine	1.95	0.46–0.79	mg/dL	H
Uric acid	6.9	2.6–5.5	mg/dL	H
Sodium (Na)	137	138–145	mmol/L	L
Potassium (K)	2.9	3.6–4.8	mmol/L	L
Chloride (Cl)	105	101–108	mmol/L	
C-reactive protein	3.79	<0.14	mg/dL	H
WBC	20,590	3,300–8,600	/µL	H
RBC	3.86	3.86–4.92	×10⁶/µL	
Hemoglobin	11.5	11.6–14.8	g/dL	L
Hematocrit	35.2	35.1–44.4	%	
Platelet count	334	158–348	×10³/µL	
MCV	91.2	84.0–98.0	fL	
MCH	29.8	28.0–33.0	pg	
MCHC	32.7	32.0–35.0	%	

**Figure 2:**
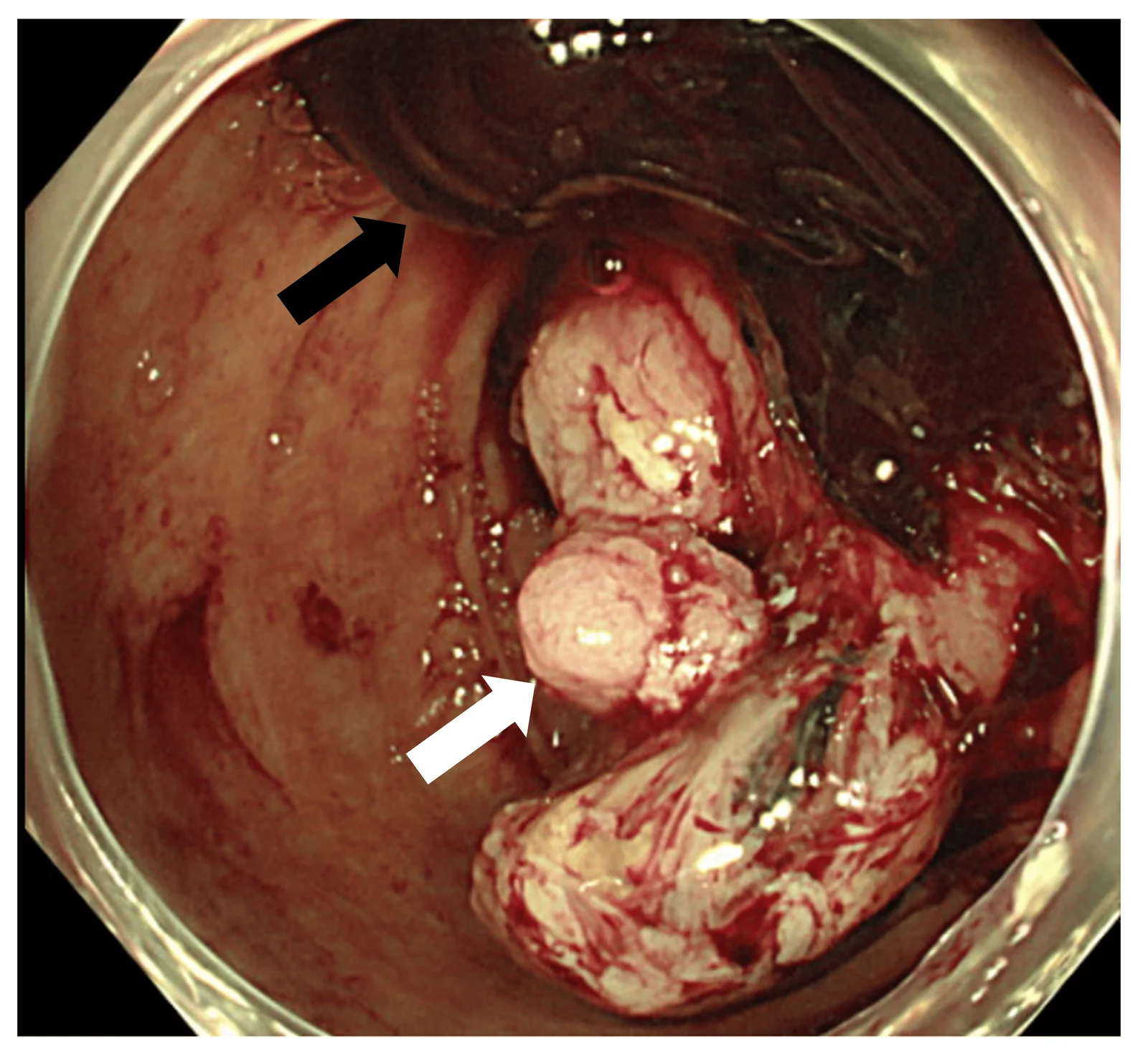
Endoscopic view of the transverse colon tumor. A circumferential, friable, and ulceroproliferative tumor (white arrow) is observed near the hepatic flexure, with an adherent blood clot (black arrow) on its surface.

Colonoscopy was performed the same day with a standard colonoscope (PCF-H290I; Olympus, Tokyo, Japan). Bowel preparation consisted of a retrograde warm-water enema (300 mL) alone, a pragmatic choice given the patient’s hemodynamic fragility and complete obstruction. During insertion, loops were reduced as needed under fluoroscopic guidance, and the transverse colon was loop-free at the time of stent deployment; scope stability was nonetheless poor, because the endoscope was pushed back whenever it was advanced toward the tumor, limiting transmission of force to the catheter tip. Colonoscopy revealed a circumferential, friable, ulceroproliferative tumor near the hepatic flexure (Figure 2) producing a near-complete stenosis that did not admit the colonoscope. The residual lumen was eccentric and only barely traversable by a guidewire, and, owing to the acute flexure angulation, its axis ran tangential to the endoscopic line of approach, so that the orifice could not be visualized fully en face.

Guidewire passage was first attempted with a 0.035-inch angled-tip hydrophilic guidewire (Jagwire; Boston Scientific, Marlborough, MA) delivered through a standard ERCP catheter (MTW Endoskopie, Wesel, Germany). Despite catheter torque, repeated re-angulation of the guidewire tip by torque, and patient repositioning, the catheter tip could not be brought coaxial with the eccentric orifice and the guidewire could not be advanced across the stricture. Exchange for a differently shaped catheter was not attempted; instead, the catheter was exchanged for a rotatable sphincterotome (ENGETSU; Kaneka Medix, Osaka, Japan).

**Figure 3:**
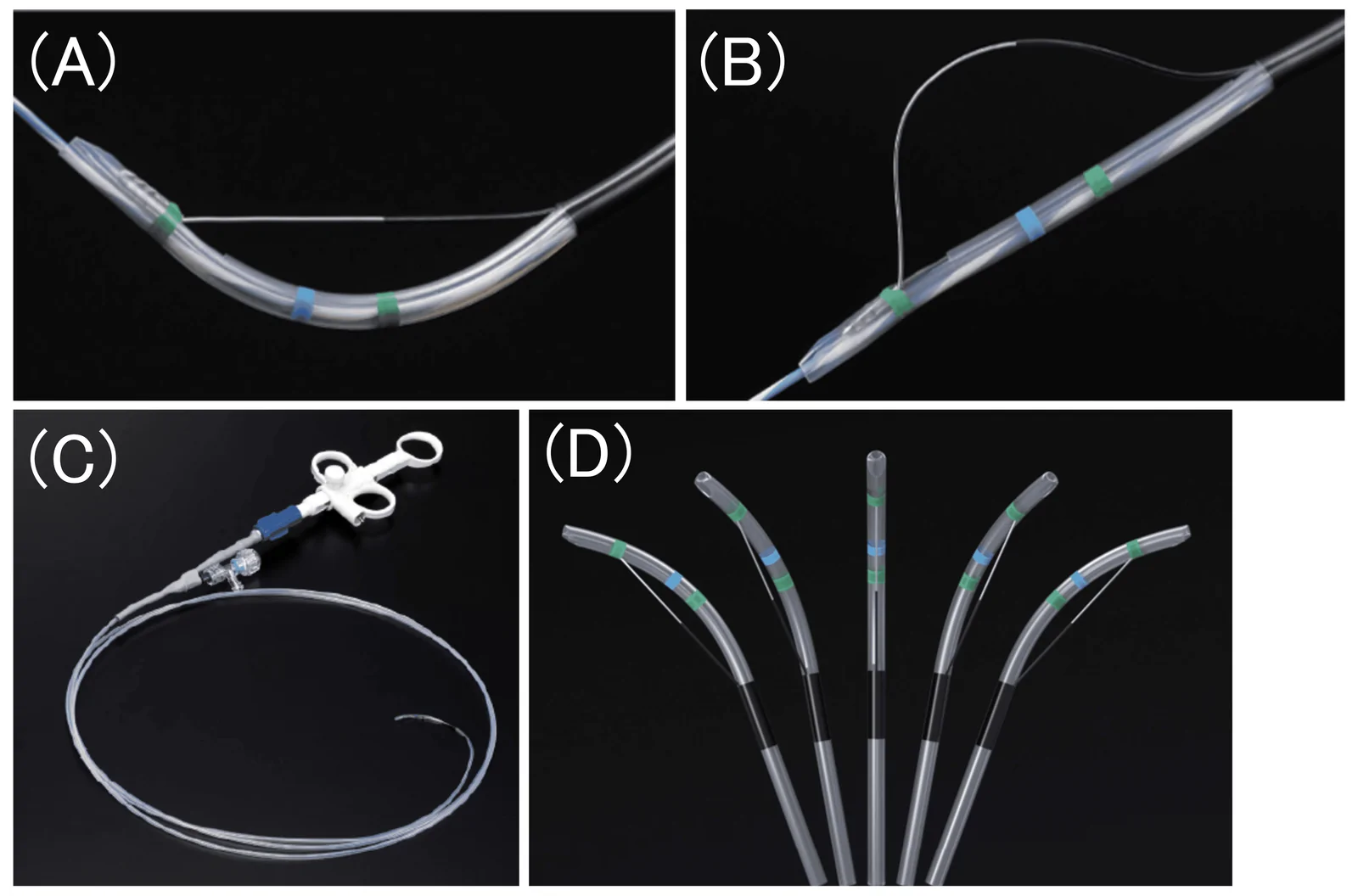
The rotatable sphincterotome and its tip-control mechanism. (A) Tip bending achieved by tensioning the cutting wire through handle manipulation. (B) Return to the straightened configuration by releasing wire tension. (C) Overview of the complete device, showing the rotatable handle assembly and catheter shaft. (D) Sequential positions of the catheter tip demonstrating 360-degree circumferential reorientation achieved by rotating the handle, illustrating the multiplanar directional control of the device.

**Figure 4:**
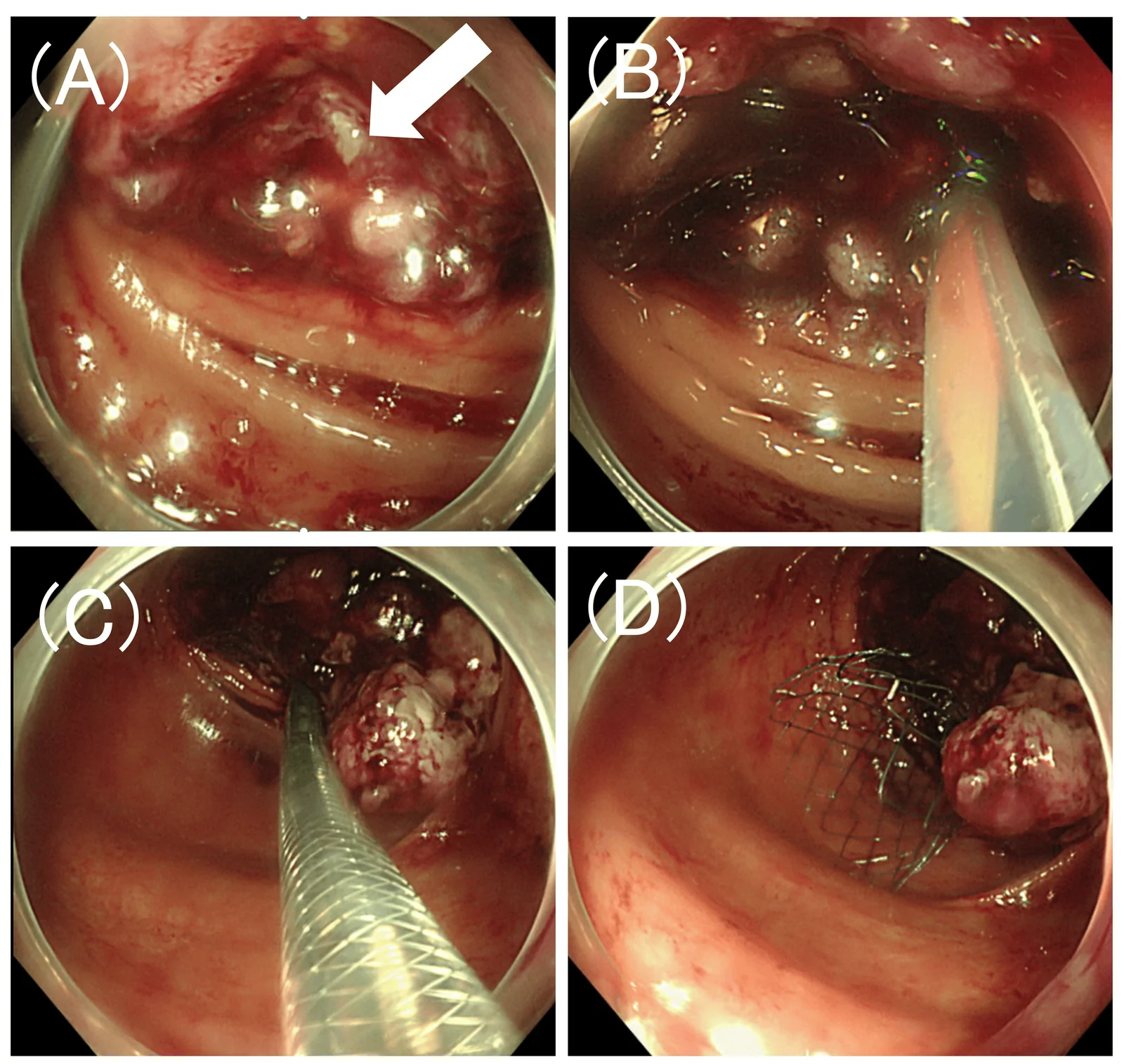
Sequential endoscopic images documenting the stepwise progression from guidewire failure to successful stent deployment. (A) En face view of the malignant stricture at the hepatic flexure. The eccentric residual luminal orifice (arrow) is deviated out of the axial plane accessible to the standard catheter, which could not be inserted coaxially despite repeated attempts and patient repositioning. (B) The rotatable sphincterotome was introduced through the working channel of the colonoscope; incremental handle rotation and tip-bending adjustment directed the catheter tip coaxially into the eccentric orifice, allowing the guidewire to be advanced through the stricture under fluoroscopic guidance. (C) A 22 mm × 120 mm uncovered Niti-S colonic self-expandable metal stent (SEMS) delivery system (Taewoong Medical, Seoul, South Korea) was advanced over the guidewire into the malignant stricture using the over-the-wire technique. (D) Fully deployed 22 mm × 120 mm uncovered Niti-S colonic SEMS covering the stricture.

**Figure 5:**
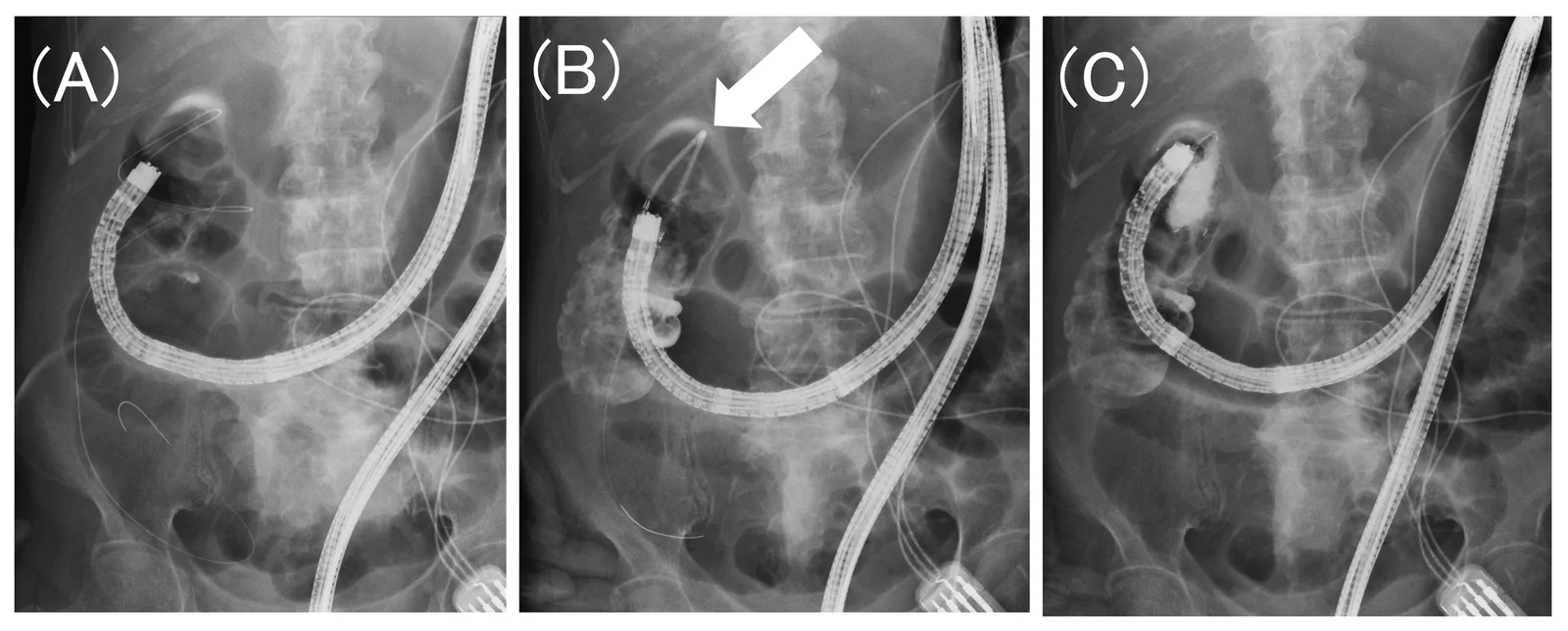
Sequential fluoroscopic images confirming guidewire passage and stent deployment at the hepatic flexure. (A) After successful use of the rotatable sphincterotome, the guidewire has traversed the malignant stricture and its tip lies well within the ascending colon. (B) The 22 mm × 120 mm uncovered Niti-S colonic self-expandable metal stent (SEMS) delivery system (Taewoong Medical, Seoul, South Korea; arrow) advanced over the guidewire across the stricture. The 120 mm length was selected to provide a 30 mm safety margin beyond the fluoroscopically measured 90 mm obstruction. (C) Fully deployed 22 mm × 120 mm uncovered Niti-S colonic SEMS at the hepatic flexure, with adequate radial expansion and complete coverage of the stricture.

The cutting wire was not connected to an electrosurgical unit and was not activated at any point; the device was used solely for mechanical tip steering, eliminating any risk of thermal injury to the colonic wall. By adjusting handle rotation and tip bending, the tip was directed toward the eccentric orifice, and the guidewire was advanced across the stricture under fluoroscopic guidance (Figures 3-5). Fluoroscopic measurement by catheter withdrawal confirmed an obstruction length of approximately 90 mm. Over the guidewire, an uncovered 22 mm × 120 mm Niti-S colonic SEMS (Taewoong Medical, Seoul, South Korea) was deployed using the over-the-wire technique under combined endoscopic and fluoroscopic guidance, fully covering the stricture; the 120 mm length provided a 30 mm margin beyond the 90 mm stenosis. Complete deployment was confirmed fluoroscopically, with immediate satisfactory fecal effluent confirming luminal patency. The total procedure time was 63 minutes and the fluoroscopy time was 23 minutes. No immediate adverse events occurred.

**Figure 6:**
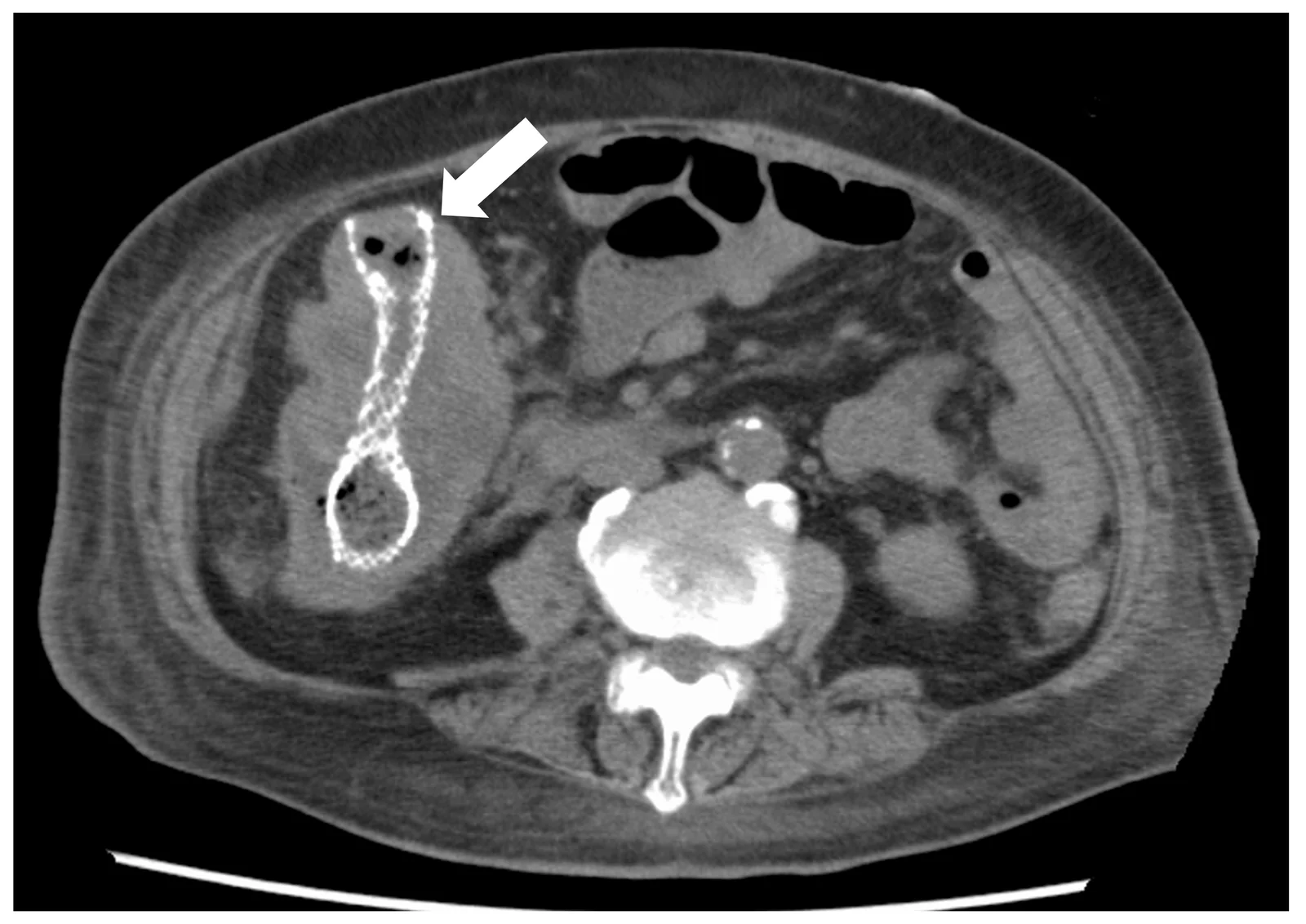
Unenhanced axial computed tomography image obtained on postprocedure day one. Unenhanced axial computed tomography (CT) image obtained on postprocedure day one, showing the uncovered Niti-S colonic self-expandable metallic stent (arrow) correctly positioned at the hepatic flexure. The stent is expanded across the malignant stricture with no evidence of migration or perforation. In direct comparison with the preprocedure CT (Figure 1), the marked proximal colonic dilatation has substantially resolved, confirming successful luminal decompression.

**Figure 7:**
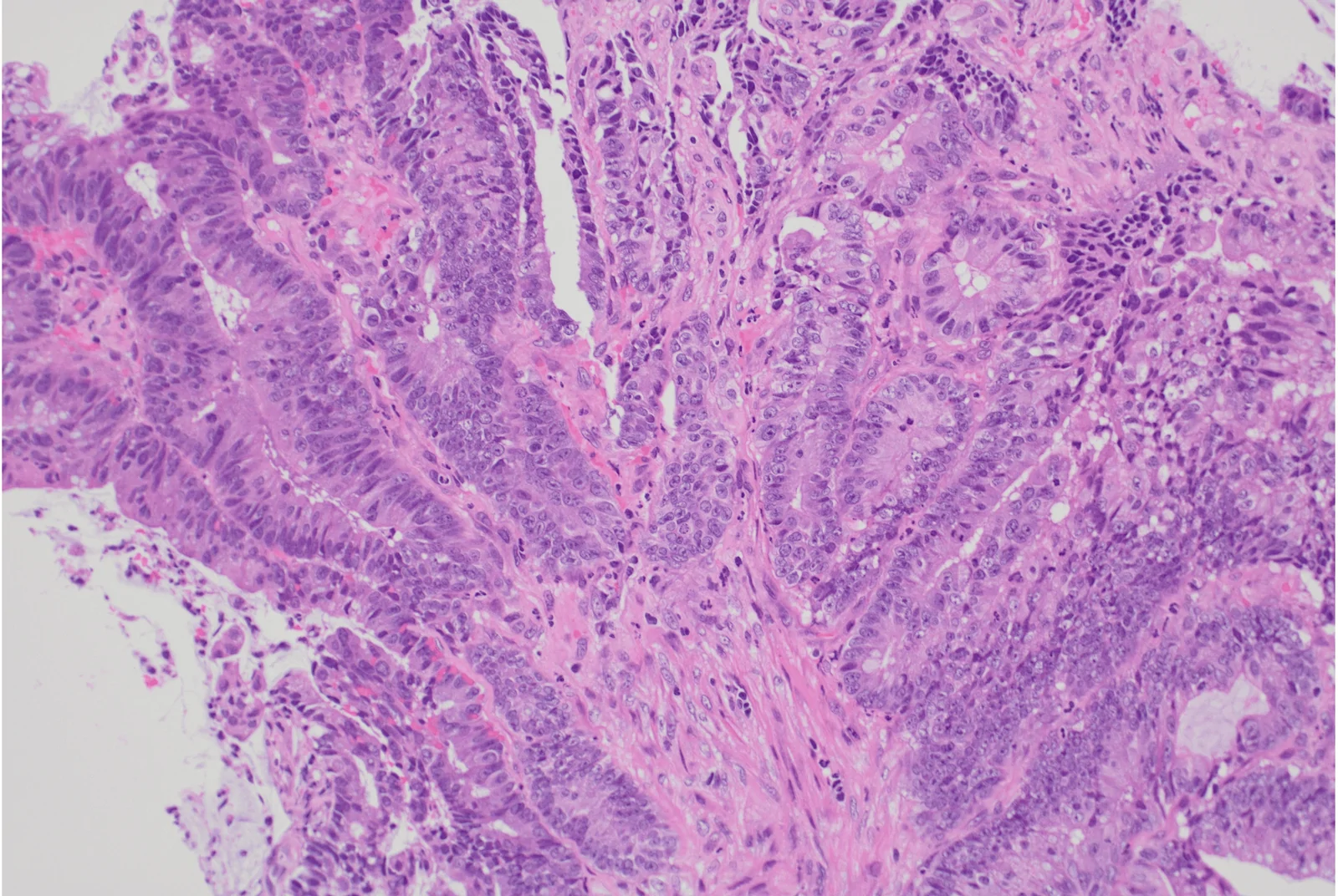
Photomicrograph of a biopsy specimen from the transverse colon tumor (hematoxylin and eosin stain; original magnification ×200). Atypical columnar epithelium proliferates in a complex, irregularly branching tubular architecture with loss of normal glandular polarity. The nuclei are markedly enlarged, hyperchromatic, and pleomorphic, ranging from elongated spindle-shaped to rounded oval forms, with stratification and focal loss of basal orientation. These features are diagnostic of well-to-moderately differentiated adenocarcinoma.

Postprocedure CT on day 1 confirmed correct stent positioning at the hepatic flexure, with adequate expansion and relief of the proximal distension (Figure 6). The patient resumed oral intake on postprocedure day 1. Biopsy confirmed well-to-moderately differentiated adenocarcinoma (Figure 7). After multidisciplinary discussion and in accordance with the wishes of the patient and her family, and given her advanced age, frailty, and the absence of radiological evidence of distant metastasis, aggressive antineoplastic therapy was not pursued, and a best-supportive-care plan was established. No recurrent obstruction occurred, and no reintervention was required. After a course of physical rehabilitation, she was transferred to a long-term care facility on postprocedure day 29 in stable condition; follow-up ended at that transfer, with no subsequent contact.

## Discussion

This case illustrates a technically demanding scenario in colonic SEMS placement: complete failure of guidewire passage through an eccentrically oriented malignant stricture at the hepatic flexure, resolved by use of a rotatable sphincterotome. Three interrelated points merit discussion: the anatomical and technical determinants of guidewire failure in right-sided colonic obstruction; the rationale and limitations of existing rescue strategies, including conventional SAGWI; and the mechanism by which multiplanar tip control overcame those limitations here.

Guidewire passage is the single most important determinant of technical success in colonic SEMS placement, yet it fails in 10-24% of procedures [5-7]. Failure is not distributed uniformly: right-sided lesions, particularly at or near the hepatic flexure, carry disproportionate risk. The hepatic flexure imposes two compounding geometric constraints. First, the acute angulation limits the range of tip deflection achievable from the working channel of a colonoscope that is itself traversing a curved corridor. Second, tumors at this location frequently produce eccentric strictures whose residual lumen deviates from the endoscopic line of approach, a configuration in which the catheter tip, even when maximally deflected, cannot be inserted coaxially. In the present case, both constraints operated simultaneously, and scope stability was further compromised because the endoscope was pushed back on advancement toward the tumor. Patient repositioning, which alters the gravitational relationship between the scope and the colonic wall, was attempted but did not resolve the geometric mismatch, consistent with prior reports indicating that repositioning alone is insufficient when the principal obstacle is catheter-tip angulation rather than scope instability [10].

The rationale for substituting a sphincterotome for a straight catheter in difficult guidewire insertion is well established: blade tension deflects the tip toward an orifice that lies off-axis from the endoscopic approach. Vázquez-Iglesias et al. first described this in the colorectal setting [11], and Zhu et al., analyzing 509 patients with malignant colorectal obstruction, provided the most rigorous evidence to date, demonstrating significant improvements in guidewire insertion success (6.9% absolute gain on intention-to-treat analysis) and technical stenting success (6.5%) with SAGWI versus conventional catheter use [10]. That study also identified sharply angulated strictures as the principal independent risk factor for SAGWI failure. Conventional sphincterotomes deflect the tip in a single plane defined by the cutting-wire axis; when the residual lumen deviates in an orthogonal plane, blade tension cannot direct the catheter toward the orifice regardless of magnitude. In anatomically complex scenarios, such as a right-sided lesion approached through a looped transverse colon, this single-plane constraint is a fundamental geometric barrier that conventional SAGWI cannot overcome [10,11,15].

A rotatable sphincterotome addresses this barrier by decoupling the direction of tip deflection from catheter orientation within the scope channel, enabling circumferential reorientation of the bending plane. In practice, this is a sequential, iterative adjustment: the operator first rotates the handle to bring the bending plane into approximate alignment with the eccentric orifice, then modulates wire tension to fine-tune tip angulation until fluoroscopy confirms coaxial engagement with the stricture lumen. This represents a change in the degrees of freedom available during guidewire insertion rather than an incremental refinement of conventional SAGWI.

It is useful to position this approach among the alternatives available for difficult colonic guidewire insertion. Angled-tip catheters provide a fixed, preformed tip angle but cannot reorient that angle circumferentially, so they fail when the orifice lies outside the plane of the preset curve. Conventional ERCP sphincterotomes deflect only in the single plane of the cutting-wire axis, the mechanistic limitation noted above. Hydrophilic angled guidewires improve trackability--indeed, we used a 0.035-inch angled hydrophilic guidewire--but a bare guidewire cannot be actively steered coaxially into an eccentric off-axis orifice and tends to buckle against the tumor surface, as occurred here; the advantage of the rotatable sphincterotome lies in providing a directable, mechanically supported tip that gives the wire a stable platform aligned with the luminal course. Thinner (pediatric) colonoscopes can aid in negotiating angulated segments and reducing looping, but the limiting problem in this case was tip-to-orifice alignment rather than scope passage or looping--the colon was loop-free at deployment--and is not addressed by a smaller scope caliber. Fluoroscopy-guided wire manipulation was employed throughout but by itself does not overcome the inability to bring the tip coaxial with an off-axis orifice. Conventional SAGWI addresses off-axis orifices but only within a single deflection plane. The specific niche of the rotatable sphincterotome is therefore the subset of difficult cases in which the eccentric orifice lies in a plane not reachable by single-plane deflection.

This class of device was originally applied in biliary and pancreatic procedures, including selective biliary cannulation in surgically altered anatomy, transpancreatic biliary sphincterotomy, and selective guidewire insertion through endoscopic ultrasound-guided hepaticogastrostomy fistulae [12-14]. The present case extends its potential utility to the colorectal setting, a context not previously described to the best of our knowledge. The transfer of technical advantage is mechanistically plausible: in both contexts the fundamental challenge is to direct a catheter tip coaxially into a narrow orifice whose axis deviates from the endoscopic line of approach, and additional rotational freedom addresses this challenge irrespective of anatomical location.

One safety consideration deserves emphasis. The colonic wall, particularly with obstructive colitis and associated mural edema as in this case, is more fragile than the bile or pancreatic duct. Sphincterotome manipulation in the colonic lumen therefore carries a non-trivial risk of mucosal laceration or perforation, especially if lateral force is inadvertently applied against the wall. In the present case, the cutting wire was never energized and was used purely for mechanical steering; three-dimensional tip control may allow gentler manipulation than conventional non-rotating sphincterotomes and could thereby reduce this risk, though this reasoning is inferential and requires prospective validation.

Several features merit comment regarding clinical decision-making. The patient’s comorbidity profile rendered surgery hazardous and removed emergency surgery as a fallback had endoscopic decompression failed, increasing the importance of first-pass technical success. The markedly elevated leukocyte count and CRP reflected the systemic inflammatory burden of complete obstruction and obstructive colitis and reinforced the need for prompt decompression. An uncovered SEMS was chosen deliberately, since covered stents carry a significantly higher migration risk in the palliative colorectal setting and are not generally recommended [8,9]. The 120 mm length provided a 30 mm margin beyond the fluoroscopically measured 90 mm stricture, consistent with standard practice.

This report has several limitations. It is a single-case experience of unknown generalizability, and no causal inference regarding the superiority of the rotatable sphincterotome over alternative rescue strategies can be drawn; the counterfactual--whether a conventional sphincterotome with further repositioning might ultimately have succeeded--cannot be assessed retrospectively. The device entails additional cost relative to a standard catheter or conventional sphincterotome, and coordinating handle rotation with wire-tension adjustment involves a learning curve. Importantly, the technique still requires a residual lumen through which a guidewire can pass and cannot overcome complete luminal obliteration; in this case, the lumen, though eccentric and severely narrowed, remained barely traversable. Follow-up was limited, ending at transfer to long-term care on postprocedure day 29, so long-term oncological and stent-related outcomes are unknown and, given the palliative intent, cannot be generalized. Prospective comparative studies, ideally a randomized controlled trial comparing conventional SAGWI with rotatable-sphincterotome-assisted guidewire insertion in technically difficult colonic obstruction, are warranted to determine whether the observed technical advantage translates into a clinically meaningful improvement at the population level.

## Conclusions

A rotatable sphincterotome may serve as a useful rescue tool for technically difficult colonic SEMS placement when conventional catheters fail to achieve guidewire passage through an eccentrically oriented or acutely angulated malignant stricture. Its capacity to reorient the bending plane circumferentially provides an additional degree of freedom for coaxial tip alignment that single-plane devices cannot. This appears to be the first reported use of a rotatable sphincterotome for colonic SEMS placement; sphincterotome-assisted guidewire insertion itself is an established technique. Further prospective studies are needed to define the role of this device within the algorithmic approach to difficult colonic stenting and to determine whether its technical advantage reduces procedural failure and complication rates.
